# Mathematical Modeling of Glutathione Status in Type 2 Diabetics with Vitamin B_12_ Deficiency

**DOI:** 10.3389/fcell.2016.00016

**Published:** 2016-03-23

**Authors:** Varun Karamshetty, Jhankar D. Acharya, Saroj Ghaskadbi, Pranay Goel

**Affiliations:** ^1^Department of Mathematics, Indian Institute of Science Education and ResearchPune, India; ^2^Department of Zoology, University of PunePune, India; ^3^Department of Biology, Indian Institute of Science Education and ResearchPune, India

**Keywords:** vitamin B_12_ deficiency, hyperhomocysteinemia, type-2 diabetes, glutathione, cysteine-block

## Abstract

Deficiencies in vitamin B_12_ and glutathione (GSH) are associated with a number of diseases including type 2 diabetes mellitus. We tested newly diagnosed Indian diabetic patients for correlation between their vitamin B_12_ and GSH, and found it to be weak. Here we seek to examine the theoretical dependence of GSH on vitamin B_12_ with a mathematical model of 1-carbon metabolism due to Reed and co-workers. We study the methionine cycle of the Reed-Nijhout model by developing a simple “stylized model” that captures its essential topology and whose kinetics are analytically tractable. The analysis shows—somewhat counter-intuitively—that the flux responsible for the homeostasis of homocysteine is, in fact, peripheral to the methionine cycle. Elevation of homocysteine arises from reduced activity of methionine synthase, a vitamin B_12_-dependent enzyme, however, this does not increase GSH biosynthesis. The model suggests that the lack of vitamin B_12_–GSH correlation is explained by suppression of activity in the trans-sulfuration pathway that limits the synthesis of cysteine and GSH from homocysteine. We hypothesize this “cysteine-block” is an essential consequence of vitamin B_12_ deficiency. It can be clinically relevant to appreciate that these secondary effects of vitamin B_12_ deficiency could be central to its pathophysiology.

## Introduction

Vitamin B_12_ (cobalamin) deficiency is a major health concern worldwide (Stabler and Allen, 2004; Stabler, 2013). Vegans, and to a lesser extent lactoovovegetarians and lactovegetarians, are at risk for developing cobalamin deficiency (Herrmann et al., 2003). Several studies have argued that vegetarianism is a possible reason for a prevalent vitamin B_12_ deficiency among Indians (Refsum et al., 2001; Antony, 2003; Stabler and Allen, 2004). Another disease, type 2 diabetes mellitus, is on the rise (Brownlee, 2005; Houstis et al., 2006; Pi et al., 2007; Hoehn et al., 2009; Leloup et al., 2009; Fisher-Wellman and Neufer, 2012; Acharya et al., 2014; Watson, 2014), with some of the fastest rates of growth in India and southeast Asia, where a considerable proportion of the population is vegetarian. This has sparked the speculation that vegetarianism may not only lead to vitamin B_12_ deficiency but also exacerbate diabetes in these parts. For example, in the Pune Maternal Nutrition Study, Yajnik et al. (2008) show that mothers with a combination of high folate and low vitamin B_12_ concentrations had children with high insulin resistance, and therefore at risk for developing diabetes later in life. The growing incidence of diabetes among Indians, as in the world, is a relatively recent phenomenon and is probably the result of lifestyle changes associated with over-nutrition. It is intriguing to ask if vegetarianism and the prevalence of vitamin B_12_ deficiency in Indians makes them particularly susceptible to the environmental insults that lead to diabetes.

One possible candidate for such a link is oxidative stress. Oxidative stress has long been associated with diabetic complications, but has only recently received attention as a possible reason for the *development* of diabetes (Houstis et al., 2006; Hoehn et al., 2009; Watson, 2014). We have recently argued that the extent of oxidative stress determines the severity with which diabetes presents itself (Kulkarni et al., 2014a,b). We have found that glutathione (GSH), which is a key cellular antioxidant, is a significant reporter of oxidative stress in diabetic patients and control subjects. We hypothesize the following: It is possible that vitamin B_12_ deficiency may be a major factor responsible for impaired GSH levels. If this stressed antioxidant defense network then succumbs to further oxidative pressure arising from, for example, the ingestion nutrients in excess or chronic inactivity, that in turn may contribute to the development of diabetes.

**Figure 1:**
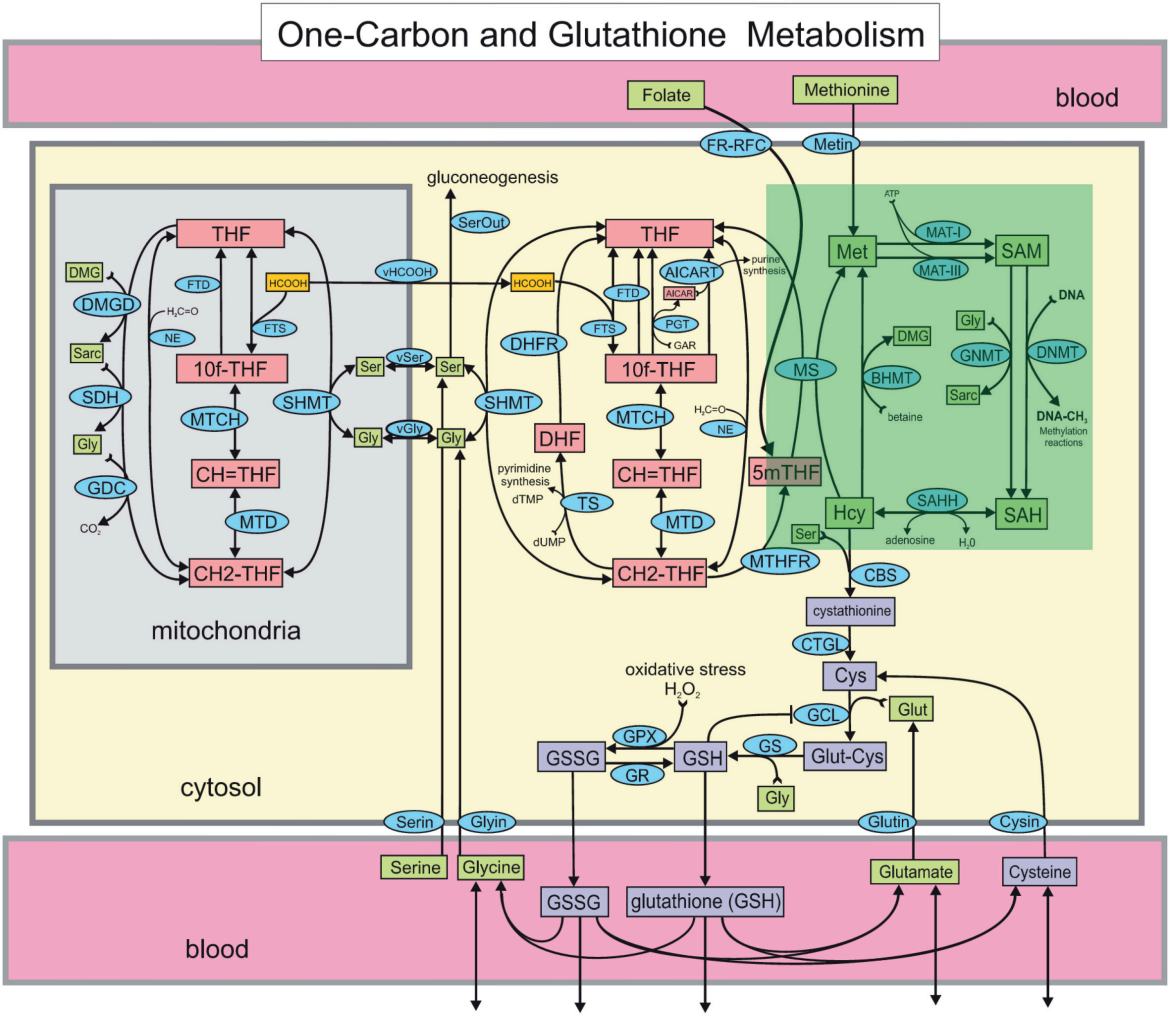
**The Reed-Nijhout model of 1-carbon and glutathione metabolism reproduced from Reed et al. (2008)**. The methionine cycle, highlighted in the green box, is comprised of the metabolites homocysteine (Hcy), methionine (Met), S-adenosylmethionine (SAM) and S-adenosylhomocysteine (SAH). The effect of vitamin B_12_ in the model is simulated by varying the reaction velocity of methionine synthase (MS), that is, $V_{max}^{MS}$. Apart from the remethylation to Met, Hcy also drives the synthesis of cystathionine via cystathionine β-synthase (CBS). Note that while the flux from Hcy to Met is unidirectional in the model, flux exchange between Hcy and SAH is bidirectional. Hcy is also upstream of glutathione, GSH and GSSG.

Cobalamins have been the subject of considerable recent investigation in the context of oxidative stress (Jacobsen, 2000; Hondorp and Matthews, 2004; Albu et al., 2012; Giustarini et al., 2014). Vitamin B_12_ is a co-enzyme for 5-methyltetrahydrofolate-homocysteine methyltransferase, also known as methionine synthase (MS). MS is responsible for the regeneration of methionine (Met) from homocysteine (Hcy) in the methionine cycle (see Figure 1). Vitamin B_12_ also acts as a co-factor in the conversion of methylmalonyl-CoA into succinyl-CoA by methylmalonyl-CoA mutase. Even a modest reduction in Vitamin B_12_ status will cause elevation of plasma homocysteine (Scott, 1999; Refsum et al., 2001). Thus, increased homocysteine levels (hyperhomocysteinemia), and increased methylmalonic acid (MMA) levels are symptoms of possible vitamin B_12_ deficiency. Total serum Hcy is therefore widely used to test for vitamin B_12_ deficiency. The test, however, is known to be limited in its specificity to detect cobalamin deficiency because Hcy levels are also elevated by other conditions. (Abbreviations used in this paper along with their complete names are listed in Glossary.)

An important theme in interpreting vitamin B_12_ deficiency is the “remethylation block hypothesis”: Vitamin B_12_ deficiency results in hyperhomocysteinemia because Hcy remethylation is “blocked” (Selhub et al., 2007). Mutations in the MTR gene, which encodes MS, could also lead to hyperhomocysteinemia (Watkins et al., 2002). A polymorphism in the methionine synthase reductase (MTRR) enzyme, responsible for maintaining adequate levels of cob(III)alamin, is known contribute to a moderate increase in Hcy levels (Gaughan et al., 2001). Individuals with a common mutation in methylenetetrahydrofolate reductase (MTHFR) could also have significantly elevated plasma homocysteine levels (Frosst et al., 1995). In particular, folate deficiency could also be a factor behind elevated Hcy levels (Kang et al., 1987; Chu and Hall, 1988; Stabler et al., 1993). Normal levels of both methylmalonic acid and total homocysteine almost certainly rule out clinically significant cobalamin deficiency (Savage et al., 1994).

However, a further examination of this hypothesis, for example using the topology of the metabolic network in Figure 1, shows there might be difficulties with this interpretation. For one, Hcy can be shuttled away to cystathionine, hence, in principle, a block in Hcy remethylation is not immediately a sufficient condition for Hcy elevation. Secondly, Hcy is also important for the synthesis of cysteine via the trans-sulfuration pathway, which leads to the synthesis of glutathione. In fact, it has been shown that in human liver cells as much as 50% of the cysteine in glutathione is derived from Hcy (Mosharov et al., 2000). The paradox of the remethylation block theory is thus that *hyperhomocysteinemia ought to be protective toward GSH, or in other words, that vitamin B_12_ deficiency actually has antioxidant benefit!*

These arguments demonstrate that models of the methionine cycle need to be examined in greater detail to determine if (i) GSH does indeed accumulate under vitamin B_12_ deficiency, (ii) if it does, how does vitamin B_12_—deficiency induced hyperhomocysteinemia affect GSH levels. It is useful to examine these questions not only from experiments but also from theoretical points of view; since this physiology is complex, mathematical models can play a significant part in unraveling the interactions.

Reed et al. have developed a detailed computational model of 1-carbon metabolism in Reed et al. (2004), Nijhout et al. (2004), Reed et al. (2006), and Deplancke and Gaskins (2002). We are using a computational model of 1-carbon metabolism and glutathione synthesis due to Reed et al. (2008). In Reed et al. (2006; See Tables 1, 2) they show that simulating a vitamin B_12_ deficiency by decreasing MS activity to 10% of normal did not lead to a significant change in Hcy. This suggests that the relationships between vitamin B_12_, Hcy, and GSH may be more complex than is suggested by a remethylation block hypothesis alone.

**Table 1:** **Kinetic parameters in the reduced model**.

$V_{max}^{bMetc}$	913.4	$k_{BHMT}^{bet}$	100.0
$V_{max}^{MS}$	500	$k_{max}^{MAT1}$	41.0
$V_{max}^{BHMT}$	2160	$k_{i}^{MAT1}$	2140.0
$V_{max}^{MAT1}$	260	$k_{max}^{MAT3}$	300.0
$V_{max}^{MAT3}$	220	$k_{i}^{MAT3}$	4030.0
$V_{max}^{GNMT}$	260	$k_{GNMT}^{sam}$	63.0
$V_{max}^{DNMT}$	180	$k_{i}^{GNMT}$	18.0
$V_{f}^{SAHH}$	320	$k_{max}^{DNMT}$	1.4
$V_{r}^{SAHH}$	4530	$k_{i}^{DNMT}$	1.4
$V_{max}^{CBS}$	420000	$k_{SAHH}^{sah}$	6.5
*k*_*bmetc*_	150	$k_{SAHH}^{hcy}$	150.0
$k_{MS}^{5mf}$	25.0	$k_{CBS}^{hcy}$	1000.0
$k_{MS}^{hcy}$	1.0	$k_{CBS}^{ser}$	2000.0
$k_{BHMT}^{hcy}$	12.0		

*Parameters used in constructing the reduced model are as in Reed et al. (2008). Time is in hours, concentrations are in μM*.

**Table 2:** **Kinetic parameters in the reduced model**.

Betaine	50
Blood methionine	30
Cytosolic serine	605
Cytosolic glycine	1300
Cytosolic glutathione disulfide	60

*The table shows the values of the constants we assigned to each of the metabolites that were originally variables in the Reed-Nijhout model. Concentrations are in μM*.

Here we examine vitamin B_12_ and GSH measured from newly diagnosed Indian diabetic patients to study any correlation between these variables. In addition, we explore the relationship between vitamin B_12_, Hcy, and GSH in greater detail using the Reed-Nijhout model. Further, we analyze the methionine cycle sub-network in the Reed-Nijhout model in order to interpret experimental data in light of model predictions.

## Materials and methods

### Experimental methods

Fasting blood samples were collected from fifty non-diabetic subjects and fifty-four diabetic patients at baseline and at follow-up visits after 4 and 8 weeks of anti-diabetic treatment, as described previously in Acharya et al. (2014). Plasma was separated from blood samples and a colistin sulfate-resistant strain of *L. leichmanii* was used to measure plasma vitamin B_12_. For further details of the protocol please see Katre et al. (2010).

The study protocol was approved by the Institutional Ethical Committee, KEM Hospital and Research Centre, Pune. Informed consent was obtained in writing from all individuals after explaining the purpose and nature of the study.

### The reed-nijhout model of 1-carbon metabolism

Here we use a computational model of 1-carbon metabolism and glutathione synthesis due to Reed et al. (2008). The version of the Reed-Nijhout model we use was encoded by Lukas Endler (available for download on the European Bioinformatics Institute's models database Endler, 2008). The full equations and parameters of the Reed-Nijhout model we work with in this paper are described in the Supplementary Material of Reed et al. (2008).

We use XPPAUT (Ermentrout, 2002) for model simulations and MATLAB (Guide, 1998) for the analysis of our data.

### The reduced methionine cycle model

**Figure 2:**
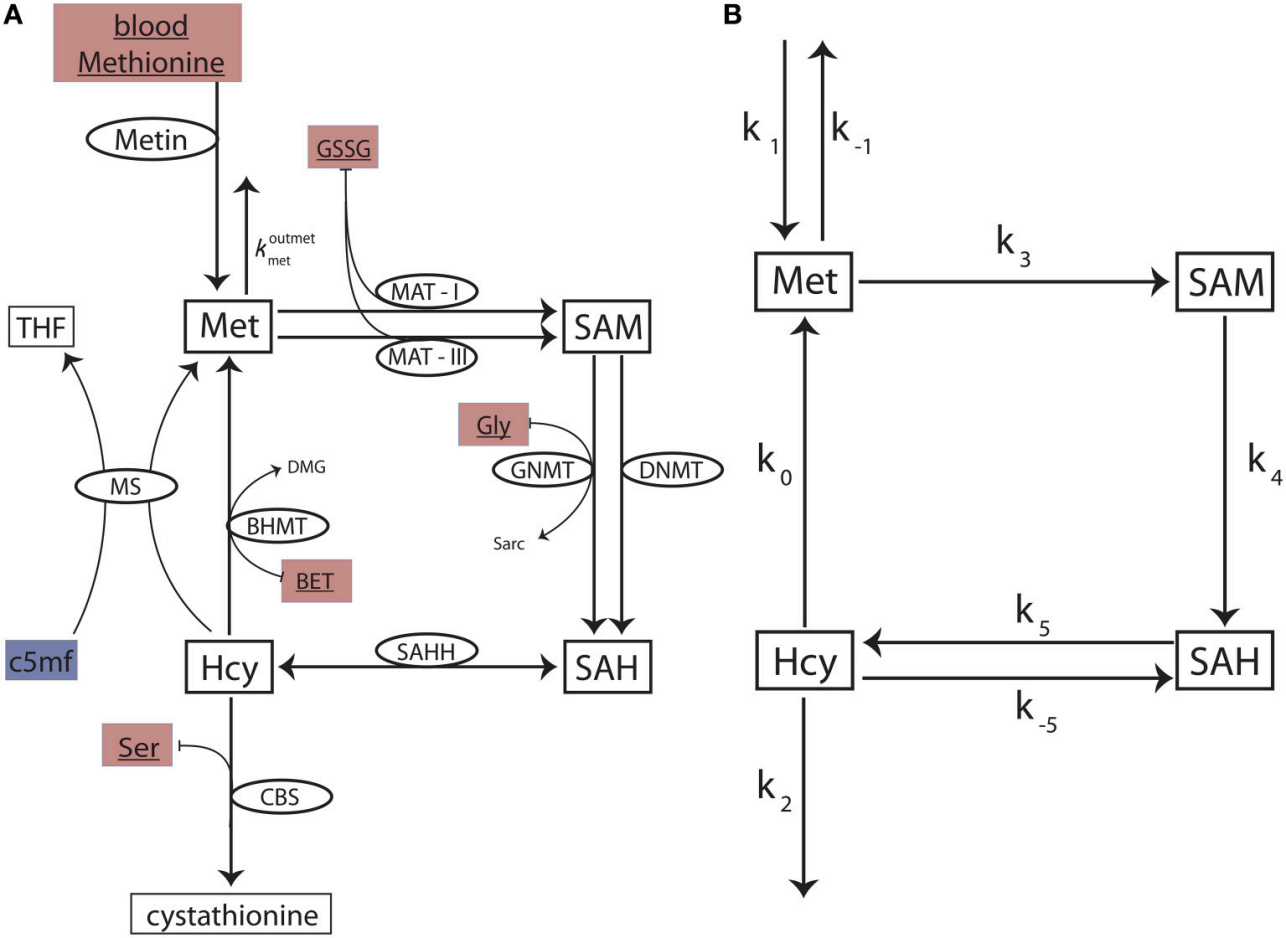
**The reduced methionine cycle model and the stylized model**. **(A)** The methionine cycle extracted from the full Reed-Nijhout model. The metabolites Met, SAM, SAH, and Hcy are variables in the model. The ellipses denote the enzymes for a reaction (flux directionality is indicated by arrows). The substrates blood methionine, GSSG, BET, Gly, and Ser (filled maroon boxes) are variables in the Reed-Nijhout model, and are taken constant in the reduced model. c5mf has been fit as a phenomenological function of $V_{max}^{MS}$ in the reduced model (shown in a blue box); see text. **(B)** A stylized model, built to capture the essential topology of the reduced model. The fluxes of the reduced model are simplified further in the stylized model.

We construct a *reduced methionine cycle model* by carefully excising the methionine cycle sub-network from the comprehensive Reed-Nijhout model (see Data Sheet 1 for full details). The reduced model (Figure 2A) is crucial in that it contains all of the components that are relevant to the dynamics of the methionine cycle even when excised from the full network. We use it to examine the essential topology and kinetics responsible for Hcy homeostasis.

### The stylized methionine cycle motif

Despite the significant reduction in size, the reduced model is not analytically tractable. To analyze the reduced methionine cycle further, we built a simplified methionine cycle *motif* (Figure 2B): An even simpler, stylized model that reflects the topology of the reduced model. We replaced the complex equations of enzyme-mediated fluxes of the reduced model (Figure 2A) with simple mass action kinetics in the stylized model (Figure 2B). See Data Sheet 1 for full details.

The mass action equations for the stylized model (Figure 2B) are:

(1) $$\begin{array}{rcl} \frac{d\;hcy}{dt} & = & k_{5}sah-\left(k_{0}+k_{2}+k_{-5}\right)hcy, \end{array}$$

(2) $$\begin{array}{rcl} \frac{d\;met}{dt} & = & k_{1}+k_{0}\;hcy-\left(k_{-1}+k_{3}\right)met, \end{array}$$

(3) $$\begin{array}{rcl} \frac{d\;sam}{dt} & = & k_{3}\;met-k_{4}\;sam, \end{array}$$

(4) $$\begin{array}{rcl} \frac{d\;sah}{dt} & = & k_{4}\;sam-k_{5}\;sah+k_{-5}\;hcy. \end{array}$$

The steady states of the metabolites are:

(5) $$\begin{array}{rcl} hcy^{*} & = & \frac{k_{1}k_{3}}{k_{3}k_{2}+k_{0}k_{-1}+k_{2}k_{-1}} \end{array}$$

(6) $$\begin{array}{rcl} met^{*} & = & \frac{k_{1}\left(k_{0}+k_{2}\right)}{k_{3}k_{2}+k_{0}k_{-1}+k_{2}k_{-1}} \end{array}$$

(7) $$\begin{array}{rcl} sam^{*} & = & \frac{k_{1}k_{3}\left(k_{0}+k_{2}\right)}{k_{4}\left(k_{3}k_{2}+k_{0}k_{-1}+k_{2}k_{-1}\right)} \end{array}$$

(8) $$\begin{array}{rcl} sah^{*} & = & \frac{k_{1}k_{3}\left(k_{0}+k_{-5}+k_{2}\right)}{k_{5}\left(k_{3}k_{2}+k_{0}k_{-1}+k_{2}k_{-1}\right)} \end{array}$$

These steady states are used to gain insight into the topology of the methionine cycle.

## Results

### Experimental results

#### Vitamin B_12_ and GSH are uncorrelated in type 2 diabetic patients

We analyzed vitamin B_12_ levels and blood GSH concentrations in Indian diabetic patients and control subjects. This data was collected as part of a clinical study conducted by us, described in Acharya et al. (2014). Briefly, we followed newly diagnosed diabetic patients over the first 8 weeks of their starting anti-diabetic therapy. We collected a wide variety of blood parameters, including GSH and other oxidative stress bio-markers, at the beginning of treatment, and at 4 and 8 weeks subsequently.

Following Selhub et al. (2007), we took a serum level of 148 pM as the threshold of diagnosis for vitamin B_12_ deficiency. While serum concentration of vitamin B_12_ generally reflects systemic vitamin B_12_ concentration, this test is not entirely specific, because other conditions, notably folate deficiency, may interfere with its interpretation. That is, low serum levels also need not immediately imply vitamin B_12_ deficiency. Nonetheless, 148 pM is taken as the threshold of diagnosis for vitamin B_12_ deficiency in epidemiological studies. We considered blood GSH level of 450 μM as the cut-off for oxidative stress (Vijayalingam et al., 1996; Acharya et al., 2014), which we have found serves to distinguish between diabetic and non-diabetic subjects fairly well.

**Figure 3:**
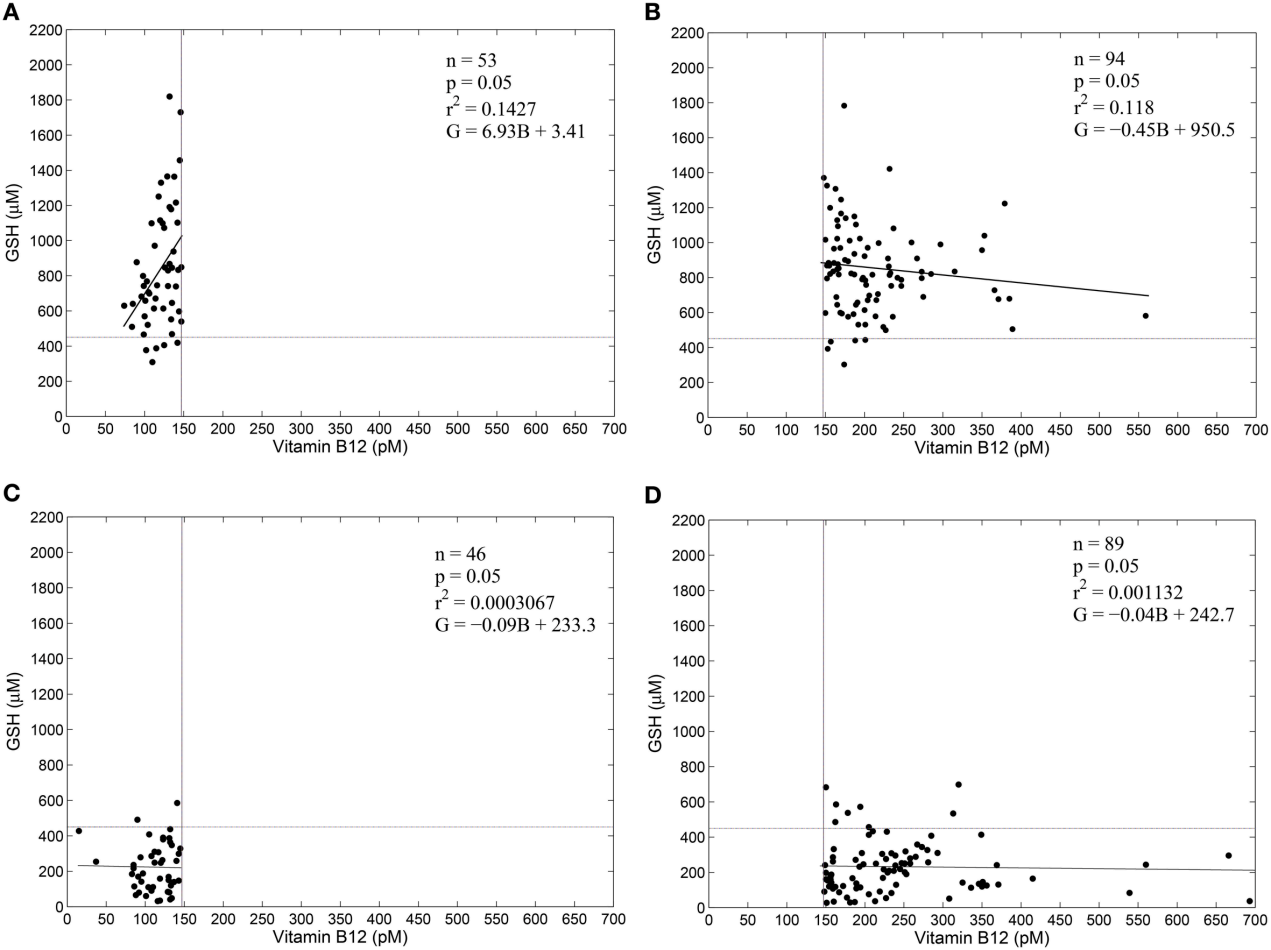
**GSH and vitamin B_12_ are weakly correlated in diabetes**. Linear regression between blood GSH and vitamin B_12_ for non-diabetic control subjects **(A,B)** and diabetic patients **(C,D)**. Individuals with serum vitamin B_12_ < 148 pM are considered vitamin B_12_–deficient (Selhub et al., 2007). Thus, **(A,C)** represent vitamin B_12_ deficiency. A GSH value of 450 μM is taken as a cut-off of oxidative stress. Notice that diabetic patients largely have GSH less than 450 μM, while control subjects have GSH greater than 450 μM. Regression statistics are indicated on the graphs directly; in the regression equations G stands for Glutathione levels in μM and B stands for vitamin B_12_ concentration in pM. Data is adapted from Acharya et al. (2014).

Figure 3 shows the results of a regression analysis of diabetic patients and control subjects, segregated into four groups based on vitamin B_12_ deficiency and oxidative stress. We find weak correlation between blood GSH and vitamin B_12_ in all the four groups. Surprisingly, there is weak correlation between blood GSH and vitamin B_12_ levels even in diabetic patients (both vitamin B_12_ deficient and otherwise). These results suggest that while oxidative stress (GSH) is a strong indicator of the diabetic status vitamin B_12_ deficiency, on the other hand, has little to do with either GSH or diabetes.

### Model results

#### The reed-nijhout model predicts GSH is protected against vitamin B_12_ deficiency

We studied the the effect of vitamin B_12_ on glutathione (GSH) using the Reed-Nijhout computational model of 1-carbon metabolism. The normal physiological value (Banerjee et al., 1990, 1997) of the reaction velocity of methionine synthase is $V_{max}^{MS}$ = 500 μM/hr. We varied $V_{max}^{MS}$ over three orders of magnitude, a very large range that includes the observed vitamin B_12_ deficiency, and studied the corresponding effect on GSH concentration. **Figure 5** shows that the effect of vitamin B_12_ on GSH is very weak in the model: Changes in $V_{max}^{MS}$ do not percolate systematically to changes in GSH.

Poor correlation between vitamin B_12_ and GSH in the experimental data is consistent with the model prediction above. This begs the question: Why do computations show that GSH relatively independent of $V_{max}^{MS}$ in the model, despite glutathione being downstream of MS activity? Although vitamin B_12_ is upstream of GSH in 1-carbon metabolism, the topology depicted in Figure 1 alone is not sufficient to anticipate how changes in $V_{max}^{MS}$ will influence GSH. If decreases in $V_{max}^{MS}$ had resulted in lowered GSH, we might have argued that decreased GSH and increased oxidative stress in diabetes may be the result of a vitamin B_12_ deficiency. On the other hand, the re-remethylation block hypothesis argues the opposite, that decreases in $V_{max}^{MS}$ are responsible for increased Hcy, and presumably GSH. The Reed-Nijhout model shows neither is true: It predicts that GSH varies largely independent of changes in vitamin B_12_. This behavior is unexpected. Below we investigate the model further to better isolate the essential component of the dynamics responsible for this feature.

### Hcy maintains homeostasis relative to $V_{max}^{MS}$ variation in the model

**Figure 4:**
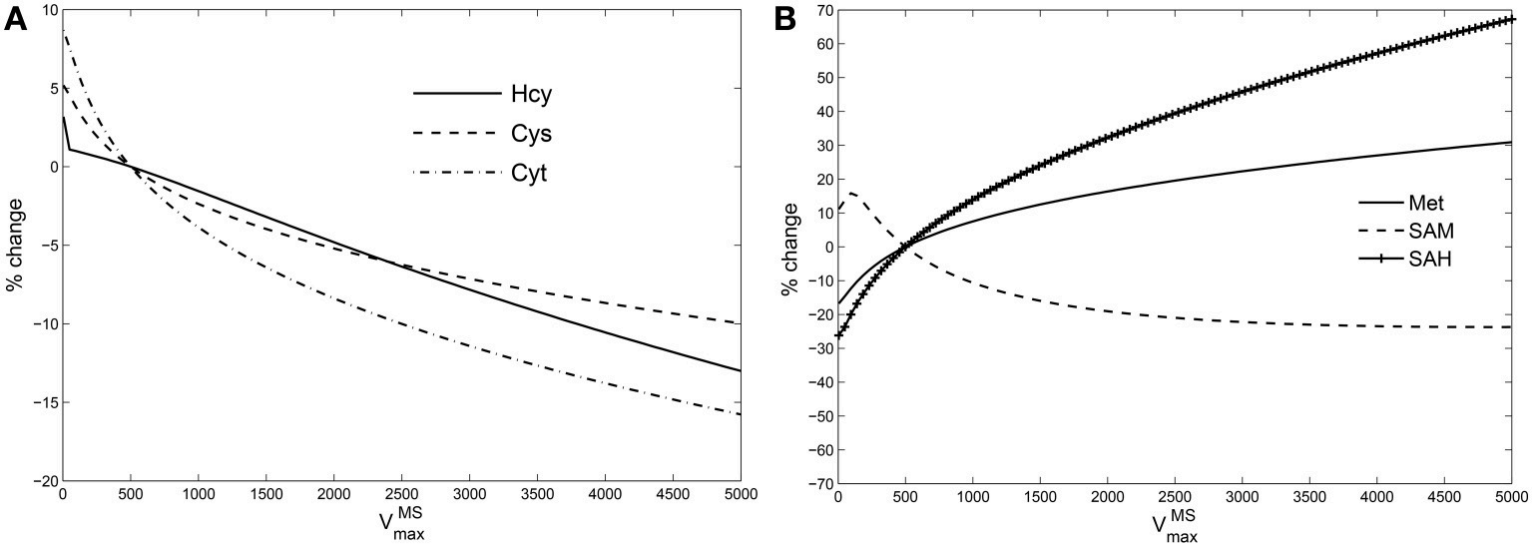
**Hcy buffers changes in vitamin B_12_**. Percentage variation in the steady state values of **(A):** species upstream of GSH leading up to Hcy, viz., cysteine (Cys), cystathionine (Cyt); **(B):** species upstream of Hcy, viz., Met, SAM, and SAH. The variations are calculated with respect to steady state value of each specie at $V_{max}^{MS}$ = 500 in the Reed-Nijhout model. It can be observed that the species shown in the figure on the right-hand side vary significantly whereas the species on the left-hand side vary by only about 15% displaying buffering against changes in $V_{max}^{MS}$.

**Figure 5:**
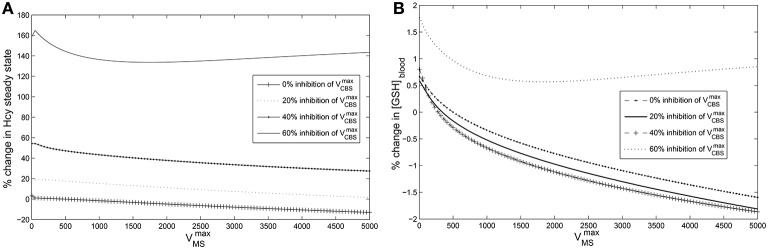
**Cysteine-block leads to hyperhomocysteinemia but prevents elevation of GSH levels**. The steady state concentrations of blood GSH and homocysteine vary with $V_{max}^{MS}$ and CBS inhibition. The steady state values of blood GSH and Hcy when $V_{max}^{MS}$ = 500 μM/Hr and $V_{max}^{CBS}$ = 420,000 μM/Hr are taken as the reference for computing % changes. **(A)** Cysteine-block leads to elevated homocysteine levels; the lower the CBS activity the higher the Hcy steady state concentrations. Hcy increases as much as 160% at 60% inhibition of $V_{max}^{CBS}$**(B)** Blood GSH varies by only 2% despite $V_{max}^{MS}$ being varied over three orders of magnitude. At 60% inhibition of $V_{max}^{CBS}$, GSH varies little. Compare to Figure 6A which shows that in the absence of cysteine-block, GSH goes up considerably with elevation in Hcy. Note that a normal diurnal variation in blood GSH is about 15%.

If GSH is independent of MS activity in the metabolic network (Figures 1, 5B), this implies that the intervening metabolites must influence this relationship significantly. We therefore systematically examined all intermediates upstream of GSH: cysteine, cystathionine, Hcy, Met, SAM, and SAH in the model while varying $V_{max}^{MS}$. Figure 4 shows that the effect of changes in $V_{m}^{MS}$ are suppressed *within* the methionine cycle, at Hcy in particular. Hcy, Cys and Cyt vary by less than 15% over 3 orders of magnitude of $V_{max}^{MS}$, while Met, SAM and SAH are seen to vary significantly more.

It appears therefore that Hcy acts as a buffer to changes in vitamin B_12_, which in turn allows for GSH to be relatively independent of vitamin B_12_. This “homeostatic” behavior of Hcy is intriguing: It runs counter to the clinical observation that vitamin B_12_ deficiency results in hyperhomocysteinemia. Moreover, the remethylation block hypothesis implies that Hcy ought to have increased significantly with lowered $V_{max}^{MS}$, which is belied by the model simulations.

Since changes in $V_{max}^{MS}$ are suppressed at the level of Hcy, examining the methionine cycle in greater detail holds the key to understanding the discrepancy between the predictions of the remethylation block hypothesis and the Reed-Nijhout model. In the following section we analyze the Reed-Nijhout model, in particular to ask: What features of the topology and the kinetics of the methionine cycle are responsible for Hcy homeostasis relative to $V_{max}^{MS}$?

### A stylized model shows a weak methionine efflux is responsible for hcy homeostasis

We are interested in the dependence of hcy^*^ on the parameter *k*_0_, which in the stylized model (described in Data Sheet 1) is representative of MS in the methionine cycle:

(9) $$\begin{array}{l} \frac{d\;hcy^{*}}{dk_{0}}=-\frac{k_{1}k_{3}k_{-1}}{{\left(k_{3}k_{2}+k_{0}k_{-1}+k_{2}k_{-1}\right)}^{2}}. \end{array}$$

The steady-state of Hcy, Equation (5) is

(10) $$\begin{array}{l} hcy^{*}=\frac{k_{1}k_{3}}{k_{3}k_{2}+k_{0}k_{-1}+k_{2}k_{-1}}. \end{array}$$

The sensitivity of hcy^*^ to changes in *k*_0_ is dependent on the values of *k*_1_, *k*_3_ and *k*_−1_; were either of these three parameters zero, hcy^*^ would be independent of changes in *k*_0_. However, *k*_1_ = 0 or *k*_3_ = 0 would result in hcy^*^ being identically zero, Equation (5). This implies that the sensitivity of Hcy steady-states to changes in *k*_0_ is dependent on the value of the parameter *k*_−1_. *k*_−1_ in the stylized model is representative of the rate constant, $k_{met}^{outmet}$, which regulates the efflux of methionine from cytosol into blood. This leads us to hypothesize that the sensitivity of Hcy to changes in $V_{max}^{MS}$ is dependent on the strength of the methionine efflux via $k_{met}^{outmet}$.

We tested the above hypothesis first in the reduced model and then in the full Reed-Nijhout model (For details please see Data Sheet 1). This behavior continues to hold in the full model: Only when the Met → blood flux parameter, $k_{met}^{outmet}$, is set to a value higher than normal, can the Hcy be seen to vary significantly with changes in $V_{max}^{MS}$.

Thus, the analysis of the stylized model reveals that *k*_−1_ acts as a control over *k*_0_, that is, how strongly $V_{max}^{MS}$ influences Hcy build up. We conclude that the essential reason Hcy maintains homeostasis over such a large range of $V_{max}^{MS}$ is a relatively low value of $k_{met}^{outmet}$ in the model. In other words, a weak methionine efflux is responsible for maintaining Hcy homeostasis in the model regardless of the availability of vitamin B_12_.

### Cysteine-block prevents hyperhomocysteinemia from elevating GSH

Next we sought to resolve the paradox: If vitamin B_12_ deficiency leads to hyperhomocysteinemia, why does GHS not also rise simultaneously?

**Figure 6:**
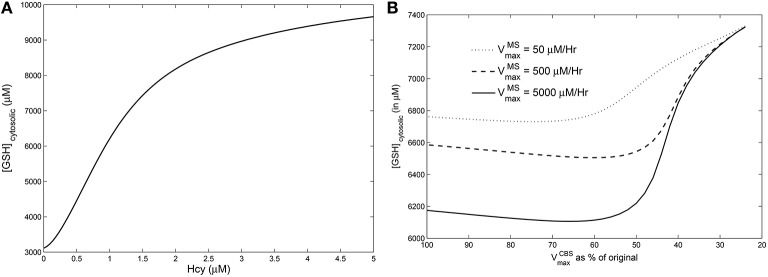
**Effects of hyperhomocysteinemia on GSH**. **(A)** Steady state values of cytosolic GSH are seen to rise by over 200% as Hcy value is turned up as a parameter. **(B)** Cytosolic GSH values are seen to change by insignificant amounts—within a range of 10% from the standard value at $V_{max}^{MS}$ = 500—with increasing inhibition in CBS. Together with the observation that inhibition of CBS activity leads to hyperhomocysteinemia, we note that cysteine-block does not simultaneously increase GSH levels.

We simulated hyperhomocysteinemia artificially (that is, we treated it as a parameter in the simulations), increasing Hcy levels to around 400% of the physiological reference value. This lead to a 200% rise in the cytosolic GSH levels (Figure 6A). However, when hyperhomocysteinemia was simulated and a cysteine-block applied (Figure 6B), it prevented a significant elevation in cytosolic GSH levels: GSH levels deviate by only about 10% from the steady-state at nominal values of $V_{max}^{MS}$ and $V_{max}^{CBS}$.

This observation leads us to conclude that vitamin B_12_ deficiency must have a *secondary effect* of inhibiting CBS activity, which in turn inhibits the flux of Hcy converting into cystathionine and protects GSH from changes in Hcy.

## Discussion

In this paper we sought to examine the relationship between vitamin B_12_ deficiency and glutathione (GSH) levels in diabetes from a theoretical standpoint. In particular, vitamin B_12_ influences the methionine cycle, of which methionine and homocysteine are major components; Hcy, in turn, influences cysteine via the trans-sulfuration pathway and GSH synthesis downstream. Clinically, hyperhomocysteinemia is associated with—and in fact, used to assess—vitamin B_12_ deficiency. Since cobalamin is a co-factor of methionine synthase, vitamin B_12_ deficiency manifests in a decreased methylation of Hcy to Met, which leads to elevated Hcy. This explanation is no doubt parsimonious, but difficulties arise in trying to reconcile hyperhomocysteinemia with GSH levels: Hcy is directly upstream of cysteine, therefore vitamin B_12_ deficiency ought to boost GSH synthesis!

We tested this prediction in diabetic patients and found that GSH levels are uncorrelated to vitamin B_12_ deficiency. We therefore turned to a detailed computational model of 1-carbon metabolism to revisit the remethylation-block hypothesis and investigate the lack of correlation between vitamin B_12_ deficiency and GSH.

The major insight from mathematical modeling is this: Hcy–Met–SAM–SAH is a *cycle*, and as such, the Hcy steady state is influenced not only by the Hcy → Met flux (including cobalamin) but also *entry and exit fluxes* of the cycle. Other authors have previously described similar ideas. For example, Liu (2005) showed that for a cyclic network the steady state does not depend on the Michaelis-Menten constants of most enzymes in the cycle, only on the branching points; reversibility can influence these “kinetic constraint conditions,” as can enzyme regulation. The network we describe does not appear to fall immediately within one of the classes described there, but our results are similar in spirit. There are essentially two exit fluxes of the methionine cycle: One is the exchange of methionine with blood, the other is the flux of homocysteine to cysteine. To ask what determines the resting concentration of Hcy it is necessary to take into account not only cobalamin (in)sufficiency but also the state of these fluxes. The steady-state concentration of Hcy is determined not only by the Hcy → Met flux but also, amongst other things, on the exit flux of Met to the blood.

The Reed model, as it stands, shows the cycle is in a mode in which the leak flux of Met to the blood is weak. The consequence of this is that Hcy maintains its level homeostatically, largely insensitive to changes in MS activity (Figure 4). To see this from the stylized model, note that Hcy flux is regulated by the product *k*_0_ × *k*_−1_, where *k*_0_ determines the Hcy → Met flux and *k*_−1_ the Met → blood flux; if *k*_−1_ is negligible *k*_0_ falls out of the picture, that is, steady-state Hcy is invariant relative to changes in vitamin B_12_. Hcy homeostasis can potentially explain why GSH is unaffected by vitamin B_12_ deficiency. However, it also raises the question why then is hyperhomocysteinemia commonly seen to occur with vitamin B_12_ deficiency. In fact, empirical evidence would seem to point to a potential weakness of the Reed-Nijhout model, that it ought to be modified to incorporate a higher Met → blood leak.

We use the Reed-Nijhout model further to confirm that if the trans-sulfuration pathway is blocked—a “cysteine-block,” as it were—GSH does not rise even if Hcy is elevated (Figure 5B). Our major insight from examining the vitamin B_12_–GSH data in conjunction with the Reed-Nijhout model is that cysteine-block explains why GSH does not increase as a result of vitamin B_12_ deficiency induced hyperhomocysteinemia. We thus hypothesize that vitamin B_12_ deficiency may have a secondary, indirect effect, one which inhibits the conversion of cystathionine to cysteine.

There is evidence in support of the cysteine-block hypothesis. For one, hyperhomocysteinemia has been known to be associated with insufficient stimulation of CBS activity (Selhub et al., 2007). SAM allosterically activates mammalian CBS 2.5-5 fold (Janosik et al., 2001), stimulating its turnover rate rather than its binding to substrate. In vitamin B_12_ deficiency, methionine block implies that SAM (driven by Met) is lowered as well and hence it is plausible CBS is less effective. In other words, the allosteric regulation of CBS by SAM may be responsible for the cysteine-block we postulate. These long-range interactions are present in the Reed-Nijhout (and reduced) models (see also Nijhout et al., 2006 for an investigation of long-range allosteric interactions between the folate and methionine cycles). Allosteric terms in the model play a role largely in “stabilizing” the steady-state concentrations of the methionine cycle substrates, especially SAM, in the face of large fluctuations in the methionine input. An interesting future direction would be to study how the allosteric regulation of CBS by SAM can be altered in the model to address cysteine-block. Finally, we chose not to include allosteric regulation explicitly in the stylized model for simplicity; it would also be interesting to ask what motif would be a simplified representation of this feature.

Other aspects of the model that are worth exploring further are the effects of compartmentalization on vitamin B_12_–Hcy–GSH metabolism, in particular, the export of Hcy directly into the blood. In fact, an alternative explanation of hyperhomocysteinemia without a concomitant increase in GSH is as follows: Excess homocysteine is exported out of the cells to avoid toxicity, and this manifests clinically as hyperhomocysteinemia. Homocysteine transport into systemic circulation enables a normal Hcy flux through the trans-sulfuration pathway, which would explain normal GSH levels in our cohort of vitamin B_12_ deficient patients. The current mathematical model does not incorporate Hcy export; therefore, further investigation is needed to establish its contribution to overall GSH homeostasis.

Further, the enzyme CBS utilizes Vitamin B_6_ as a co-factor in the conversion of Hcy to cystathionine. It is even plausible that some of the effects typically ascribed to vitamin B_12_ deficiency are, in fact, related to a vitamin B_6_ deficiency. It could be useful to distinguish between cysteine-block that arises from a deficiency of vitamin B_12_ or vitamin B_6_. In the present study we did not directly measure Hcy in the subjects. A promising direction for further study is to investigate clinically to what extent is hyperhomocysteinemia is dependent on cysteine-block. We thus believe the reinterpretation of the physiology of vitamin B_12_ deficiency that accounts for cysteine-block has several implications for clinical studies and drug discovery.

Finally, we comment on the interrelatedness of vegetarianism, vitamin B_12_ deficiency and diabetes. Had poor vitamin B_12_ levels been the reason for susceptibility to diabetes, we would have expected GSH levels to be poor in diabetic patients with vitamin B_12_ deficiency. However, both experimental data and modeling belie this: GSH is rather unaffected by vitamin B_12_ levels. Here it is also useful to note that vegetarianism, and any concomitant vitamin B_12_ deficiency, have presumably been around for several centuries in the Indian subcontinent, while the growth of diabetes is relatively recent in the last few decades. This is thus additional circumstantial evidence that while vitamin B_12_ deficiency is strongly associated with vegetarianism, neither is likely to be the major reason for the increased incidence of diabetes. On the other hand, our results could be important to the pharmacology of vitamin B_12_ supplementation, and its interaction with cellular antioxidant defense pathways.

## Author contributions

JA was responsible for collecting samples from patients and measuring their B_12_ and GSH levels. SG provided the necessary guidance in collecting these samples and measuring the B_12_ and GSH levels, along with providing the necessary lab equipment. PG, SG, and VK designed and conducted the analysis. PG guided VK in working with the mathematical model and running simulations of the stylized model. PG and VK were responsible for writing the manuscript.

### Conflict of interest statement

The authors declare that the research was conducted in the absence of any commercial or financial relationships that could be construed as a potential conflict of interest.

## Figures and Tables

**Table:** 

**Enzyme names, their acronyms and Enzyme Commission (EC) numbers**.		
MAT1	Methionine adenosyl transferase I	EC 2.5.1.6
MAT3	Methionine adenosyl transferase III	EC 2.5.1.6 (iso-enzyme of MAT1)
GNMT	Glycine N-methyltransferase	EC 2.1.1.20
DNMT	DNA-methyltransferase	2.1.1.37
SAAH	S-adenosylhomocysteine hydrolase	3.3.1.1
MS	Methionine synthase	2.1.1.13
BHMT	Betaine-homocysteine methyltransferase	2.1.1.5
CBS	Cystathione β-synthase	4.2.1.22

**Table:** 

**Variable names and acronyms**.		
Met	Methionine
SAM	S-adenosylmethionine
SAH	S-adenosylhomocysteine
Hcy	Homocysteine
cyt	Cystathionine
GSH	Glutathione
c5mf	Cytosolic 5-methyletetrahydrofolate

## Glossary

The complete names of the enzymes and variables indicated by abbreviations in this paper are as follows:
