# Supplementary material for: Mathematical Modeling of Glutathione Status in Type 2 Diabetics with Vitamin B12 Deficiency
Source: Front Cell Dev Biol. 2016 Mar 23;4:16. doi: 10.3389/fcell.2016.00016 (PMC4803754; doi:10.3389/fcell.2016.00016)
Supplement: Supplementary file 1 [file DataSheet1.pdf]

Supplementary Information

Varun Karamshetty, Jhankar Acharya, Saroj Ghaskadbi and Pranay Goel

January 28, 2016

In this Supplementary Information we give a detailed description of the construction and analysis of the mathematical models used in the main paper. The *reduced methionine cycle model* and the *stylized methionine cycle motif* were used to gain insight into the topology of the methionine cycle; the insights in turn were verified against the Reed-Nijhout model.

The Reed-Nijhout model of 1-carbon metabolism

We use the computational model of 1-carbon metabolism and glutathione synthesis due to Reed et al. (Reed et al., 2008). The version of the Reed-Nijhout model we used was encoded by Lukas Endler (available for download on the European Bioinformatics Institute's models database (Endler, 2008)).

The Reed-Nijhout-Endler model contains a feeding rhythm: parameters named *breakfast, lunch, dinner* and *fasting* were used to describe the relative levels of amino acids in the blood by using a time-step function named *aa_input*. We removed the daily feeding rhythm when using this model for our computations.

The equations and parameters of the Reed-Nijhout model (Fig. 1) we work with in this paper are described in the Supplementary Material of (Reed et al., 2008).

Construction of the reduced methionine cycle model

While we would ideally like to study the methionine cycle by itself, it is not immediately clear how to decouple the methionine cycle from the full network. Hence we show that a *reduced methionine cycle model* can be constructed by carefully excising the methionine cycle sub-network from the comprehensive Reed-Nijhout model.

Fig. 2a identifies the methionine cycle sub-network – the metabolites Hcy, Met, SAM and SAH – that we are interested in isolating. The corresponding

equations from the Reed-Nijhout model are reproduced here for completeness:

$$\frac{d\ hcy}{dt} = J_{SAHH} - J_{MS} - J_{BHMT} - J_{CBS}, \tag{1}$$

$$\frac{d\ met}{dt} = J_{bMetc} + J_{MS} + J_{BHMT} - J_{MAT1} - J_{MAT3}, \tag{2}$$

$$\frac{d\ sam}{dt} = J_{MAT1} + J_{MAT3} - J_{GNMT} - J_{DNMT}, \tag{3}$$

$$\frac{d\ sah}{dt} = J_{GNMT} - J_{DNMT} - J_{SAHH}, \tag{4}$$

where J_{MS} stands for the reaction velocity corresponding to the enzyme MS. The various reaction velocities are defined as:

$$J_{SAHH} = \left(\frac{V_f^{SAHH} \times sah}{K_{SAHH}^{sah} + sah}\right) - \left(\frac{V_r^{SAHH} \times hcy}{K_{SAHH}^{hcy} + hcy}\right)$$

$$J_{MS} = \frac{V_{max}^{MS} \times c5mf \times hcy}{(K_{MS}^{5mf} + c5mf)(K_{MS}^{hcy} + hcy)}$$

$$J_{BHMT} = e^{-0.0021(sam+sah)} e^{0.0021(102.6)} \left(\frac{V_{max}^{BHMT} \times hcy \times BET}{(K_{BHMT}^{hcy} + hcy)(K_{BHMT}^{BET} + BET)}\right)$$

$$J_{CBS} = \left(\frac{V_{max}^{CBS} \times hcy \times cser}{(K_{CBS}^{hcy} + hcy)(K_{CBS}^{ser} + cser)}\right)\left(\frac{(1.086)(sam+sah)^2}{(30)^2 + (sam+sah)^2}\right)$$

$$J_{bMetc} = \left(\frac{V_{max}^{bMetc} \times bmet}{K_{bMetc} + bmet}\right) - K_{met}^{outmet}$$

$$J_{MAT1} = \left(\frac{V_{max}^{MAT1} \times met}{K_{max}^{MAT1} + met}\right)\left(0.23 + (0.8)e^{-(0.0026)(sam)}\right)\left(\frac{K_i^{MAT1} + 66.71}{K_i^{MAT1} + cgsg}\right)$$

$$J_{MAT3} = \left(\frac{V_{max}^{MAT1} \times (met)^{1.21}}{K_{max}^{MAT1} + (met)^{1.21}}\right)\left(1 + \frac{(7.2)(sam)^2}{(K_a^{MAT3})^2 + (sam)^2}\right)\left(\frac{K_i^{MAT3} + 66.71}{K_i^{MAT3} + cgsg}\right)$$

$$J_{GNMT} = \left(\frac{V_{max}^{GNMT} \times sam \times cgly}{(K_{GNMT}^{sam} + sam)(K_{GNMT}^{gly} + cgly)}\right)\left(\frac{1}{1 + \frac{sah}{K_i^{GNMT}}}\right)\left(\frac{4.8}{0.35 + c5mf}\right)$$

$$J_{DNMT} = \frac{V_{max}^{DNMT} \times sam}{K_{max}^{DNMT}\left(1 + \frac{sah}{K_i^{DNMT}}\right) + sam}$$

Parameter values (Reed et al., 2008) are listed in Table 1.

We begin by noting that the "rest" of the network couples to the methionine cycle through the following variables: blood methionine, betaine (BET), glycine (Gly), serine (Ser), glutathione disulfide (GSSG) and 5-methyltetrahydrofolate (c5mf). That is, the kinetics of the metabolites in the methionine cycle is linked to the full network through these six quantities.

However, when we examined these "external variables" in the Reed-Nijhout model we noted that BET, Gly, Ser and GSSG changed very little with V_{max}^{MS}, Fig. 7a. This suggests that a good approximation is to treat BET, Gly, Ser and GSSG as roughly constant, i.e. as parameters in the reduced model. We build the reduced model by setting these variables to the constants shown in Table 2.

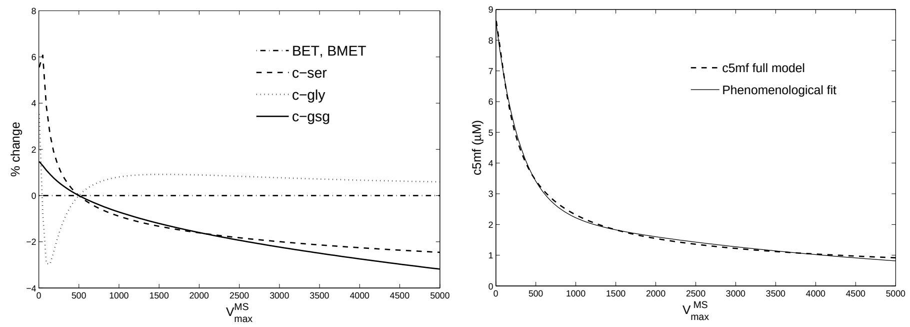

(a) (b)

Figure 7: **The approximations underlying the methionine cycle model reduction.** (a) Variation in the steady state values of BET, blood methionine (bMet), serine, glycine and glutathione disulfide with changes in V_{max}^{MS} in the full Reed-Nijhout model. It can be observed that these species vary by only about 5% even with V_{max}^{MS} varying over 3 orders of magnitude. Since the variation in these species is negligible we consider them to be constants in the reduced methionine cycle model. (b) Variation in c5mf steady-state with changing in V_{max}^{MS}. c5mf varies considerably with V_{max}^{MS}. We used a phenomenological fit of c5mf as a function of V_{max}^{MS} to describe the steady-state of c5mf in the reduced model.

On the other hand, c5mf varies substantially with V_{max}^{MS}(Fig. 7b). This is because MS, apart from playing a role in the remethylation of Hcy to Met, also acts as an enzyme in the conversion of 5-methyltetrahydrofolate (c5mf) to THF. Fig. 7b shows that the steady state value of c5mf falls with increased activity of MS. We found a good phenomenological fit of the variation of c5mf with V_{max}^{MS} to be:

$$c5mf\left(V_{max}^{MS}\right) = 6.141e^{-0.003301V_{max}^{MS}} + 2.491e^{-0.002233V_{max}^{MS}}.$$

as can be seen in Fig. 7b.

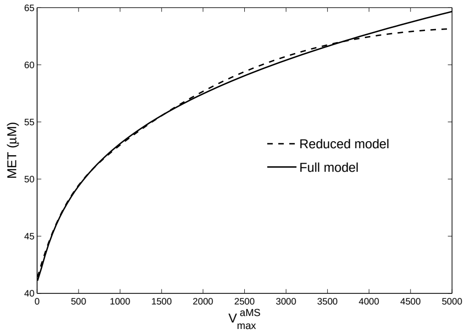

(a)

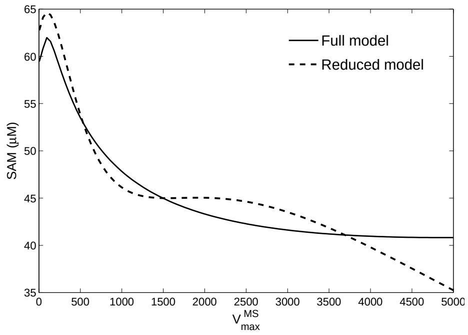

(b)

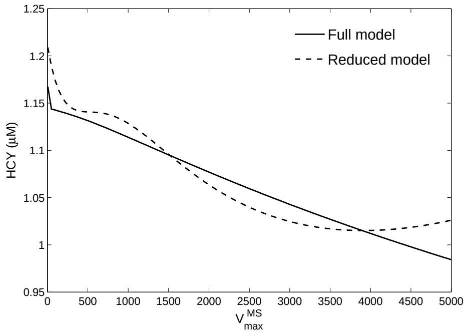

(c)

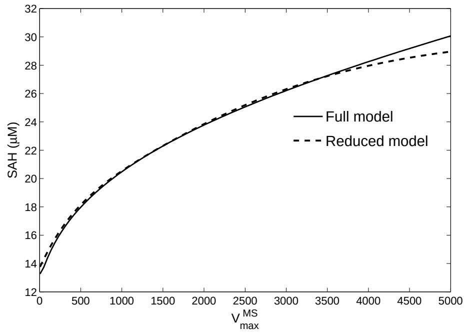

(d)

Figure 8: **Comparison of simulations from the reduced model and the full Reed-Nijhout model.** Steady state values of **(a)** Met, **(b)** SAM, **(c)** Hcy, and **(d)** SAH calculated using the reduced model as compared with the steady state values calculated using the Reed-Nijhout model. It can be observed that the reduced model closely approximates the function of the methionine cycle in the Reed-Nijhout model.

The Fig. 8 above shows that the reduced model is a reasonably good approximation of Reed-Nijhout model in modeling the function of the methionine cycle. Fig. 9 gives a numerical perspective of the information shown in Fig. 8. The maximum deviation from the Reed-Nijhout model is only by about 14%, further strengthening the validity of our reduced model.

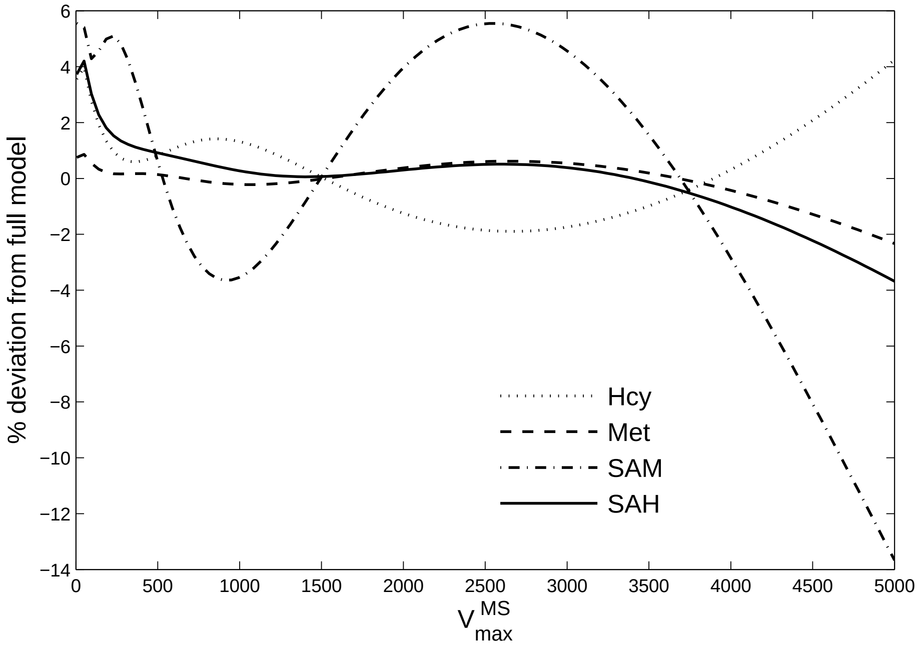

Figure 9: **Comparison of simulations from the reduced model and the full Reed-Nijhout model.** Deviation, in percentages, in the steady state values of Hcy, Met, SAM and SAH as calculated using the reduced model with respect to the steady state values calculated using the Reed-Nijhout model. It can be observed that the calculations from the reduced model deviate from those of the full well within the significant levels.

These approximations allow us to decouple the methionine cycle from the full network and construct a stand-alone reduced methionine cycle model. We verified that the reduced model displays steady-state dynamics that closely approximate the full model dynamics, Fig. 8.

Construction of the stylized methionine cycle motif

To analyze the reduced methionine cycle further, we employed the following strategy: We built a simplified methionine cycle *motif* (Fig. 2b) – an even simpler, stylized model that reflects the topology of the reduced model.

The complex equations of enzyme-mediated fluxes of the reduced model (Fig. 2a) were replaced with simple mass action kinetics in the stylized model (Fig. 2b). For example, the constant flux of methionine from blood into the cytosol is shown as a flux with rate k_1 in the stylized model. The methionine flux from the cytosol into the blood, regulated by the rate constant k_{met}^{outmet}, is assumed to be regulated by the rate constant k_{-1} in the stylized model. Fluxes due to enzymes MAT1 and MAT3, that regulate the conversion of Met

into SAM, are clubbed together and simplified to be shown as a single flux regulated by the rate constant k_3 in the stylized version. Fluxes due to enzymes GNMT and DNMT, that regulate the conversion of SAM into SAH, are also clubbed together and simplified to be shown as a single flux regulated by the rate constant k_4 in the stylized version. SAHH, which regulates the interconversion between Hcy and SAH in the reduced model, is replaced by the rate constants: k_5 to regulate the conversion from SAH to Hcy, and k_{-5} to regulate the backward conversion from Hcy to SAH in the stylized model. The rate constant k_2 in the stylized model replaces the rate constant regulating the conversion of Hcy into the cystathionine residues. Fluxes due to enzymes MS and BHMT, that regulate the remethylation of Hcy into Met, are clubbed together and simplified to be shown as a single flux regulated by the rate constant k_0 in the stylized model.

A weak methionine efflux explains the homocysteine homeostasis

We used the reduced methionine cycle model and the full Reed-Nijhout model to test the hypothesis that the sensitivity of Hcy to changes in V_{max}^{MS} is dependent on the strength of the methionine efflux via k_{met}^{outmet}. In Fig. 10 we show evidence that the models that support the hypothesis and thus, provide an explanation to the homeostatic behavior of Hcy.

We varied the value of k_{met}^{outmet} in the reduced model over three orders of magnitude and observed the changes in Hcy with respect to V_{max}^{MS} (Fig. 10a). At lower values of k_{met}^{outmet}, Hcy is almost unaffected by changes in V_{max}^{MS} whereas for higher values of k_{met}^{outmet}, Hcy varies significantly with V_{max}^{MS}. In vitamin B_{12} deficient scenario ($V_{max}^{MS} < 500$ μM/Hr), a larger value of k_{met}^{outmet} can be seen to lead to a higher steady state of Hcy resulting in hyperhomocysteinemia-like conditions.

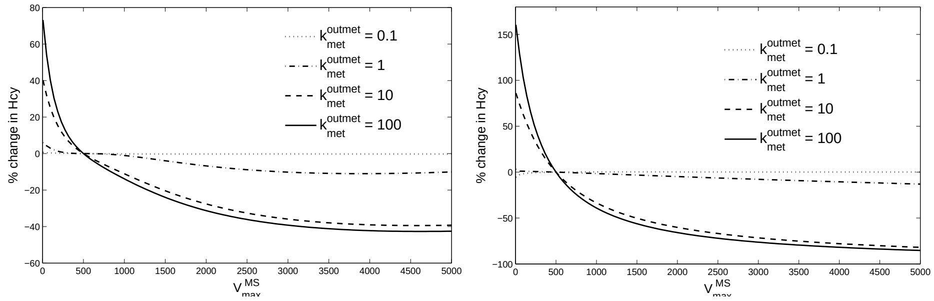

(a) (b)

Figure 10: **Steady-states of Hcy are affected by k_{met}^{outmet}.** (a) Variation in the Hcy steady states, in the reduced model, plotted against V_{max}^{MS}. (b) Variation in the Hcy steady states, in the Reed-Nijhout model, plotted against V_{max}^{MS}. Hcy steady state at $V_{max}^{MS} = 500$, for each value of k_{met}^{outmet}, is considered the baseline value for calculating the variation. Please note that the variations in percentages, depicted on the vertical axes, are different for both the figures. The effect of k_{met}^{outmet} on the variations of Hcy is more pronounced in the full Reed-Nijhout model than in the reduced model. This confirms our prediction that k_{met}^{outmet} controls the Hcy buffering capacity. Higher value of k_{met}^{outmet} leads to a larger variation in the Hcy steady-states.

References

1. Reed, M.C., Thomas, R.L., Pavisic, J., James, S.J., Ulrich, C.M., Nijhout, H.F.. A mathematical model of glutathione metabolism. *Theor Biol Med Model* 2008;**5**:8.

2. Endler, L.. European Bio-informatics Institute Bio-models Database. `http://www.ebi.ac.uk/biomodels-main/BIOMD0000000268`; 2008. [Online].
